# Linking Human Diseases to Animal Models Using Ontology-Based Phenotype Annotation

**DOI:** 10.1371/journal.pbio.1000247

**Published:** 2009-11-24

**Authors:** Nicole L. Washington, Melissa A. Haendel, Christopher J. Mungall, Michael Ashburner, Monte Westerfield, Suzanna E. Lewis

**Affiliations:** 1 Life Sciences Division, Lawrence Berkeley National Laboratory, Berkeley, California, United States of America; 2 Institute of Neuroscience, University of Oregon, Eugene, Oregon, United States of America; 3 Department of Genetics, University of Cambridge, Cambridge, United Kingdom; National Cancer Institute/NIH, United States of America

## Abstract

Scientists and clinicians who study genetic alterations and disease have traditionally described phenotypes in natural language. The considerable variation in these free-text descriptions has posed a hindrance to the important task of identifying candidate genes and models for human diseases and indicates the need for a computationally tractable method to mine data resources for mutant phenotypes. In this study, we tested the hypothesis that ontological annotation of disease phenotypes will facilitate the discovery of new genotype-phenotype relationships within and across species. To describe phenotypes using ontologies, we used an Entity-Quality (EQ) methodology, wherein the affected entity (E) and how it is affected (Q) are recorded using terms from a variety of ontologies. Using this EQ method, we annotated the phenotypes of 11 gene-linked human diseases described in Online Mendelian Inheritance in Man (OMIM). These human annotations were loaded into our Ontology-Based Database (OBD) along with other ontology-based phenotype descriptions of mutants from various model organism databases. Phenotypes recorded with this EQ method can be computationally compared based on the hierarchy of terms in the ontologies and the frequency of annotation. We utilized four similarity metrics to compare phenotypes and developed an ontology of homologous and analogous anatomical structures to compare phenotypes between species. Using these tools, we demonstrate that we can identify, through the similarity of the recorded phenotypes, other alleles of the same gene, other members of a signaling pathway, and orthologous genes and pathway members across species. We conclude that EQ-based annotation of phenotypes, in conjunction with a cross-species ontology, and a variety of similarity metrics can identify biologically meaningful similarities between genes by comparing phenotypes *alone*. This annotation and search method provides a novel and efficient means to identify gene candidates and animal models of human disease, which may shorten the lengthy path to identification and understanding of the genetic basis of human disease.

## Abstract 2

A novel method for quantifying the similarity between phenotypes by the use of ontologies can be used to search for candidate genes, pathway members, and human disease models on the basis of phenotypes alone.

## Author Summary

Model organisms such as fruit flies, mice, and zebrafish are useful for investigating gene function because they are easy to grow, dissect, and genetically manipulate in the laboratory. By examining mutations in these organisms, one can identify candidate genes that cause disease in humans, and develop models to better understand human disease and gene function. A fundamental roadblock for analysis is, however, the lack of a computational method for describing and comparing phenotypes of mutant animals and of human diseases when the genetic basis is unknown. We describe here a novel method using ontologies to record and quantify the similarity between phenotypes. We tested our method by using the annotated mutant phenotype of one member of the Hedgehog signaling pathway in zebrafish to identify other pathway members with similar recorded phenotypes. We also compared human disease phenotypes to those produced by mutation in model organisms, and show that orthologous and biologically relevant genes can be identified by this method. Given that the genetic basis of human disease is often unknown, this method provides a means for identifying candidate genes, pathway members, and disease models by computationally identifying similar phenotypes within and across species.

## Introduction

**Figure 1:**
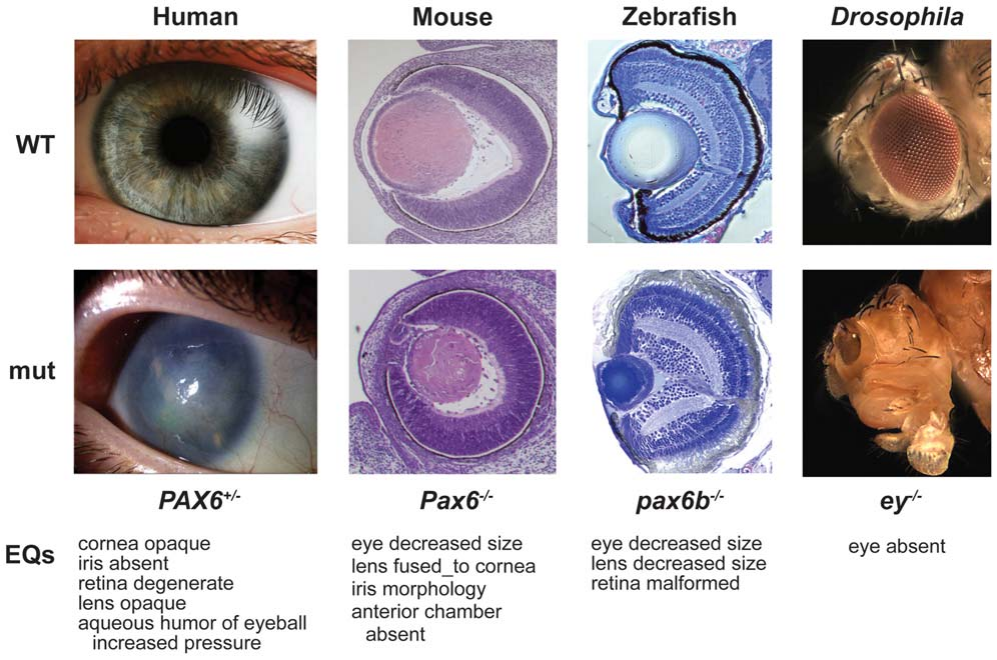
Representation of phenotypes. Phenotypes of wild-type (top) and *PAX6* ortholog mutations (bottom) in human, mouse, zebrafish, and fly can be described with the EQ method. EQ annotations of the abnormal phenotypes are listed below each set of images per organism. Note that the anatomical entities are from ssAOs and qualities are from the PATO ontology. These *PAX6* phenotypes have been described textually as follows. Human mutations may result in aniridia (absence of iris), corneal opacity (aniridia-related keratopathy), cataract (lens clouding), glaucoma, and long-term retinal degeneration. For mouse, the mutants exhibit extreme microphthalmia with lens/corneal opacity and iris abnormality, and there is a large plug of persistent epithelial cells that remains attached between the cornea and the lens. For zebrafish, the mutants express a variable and modifiable phenotype that consists of decreased eye size, reduced lens size, and malformation of the retina. Drosophila *ey* (a *PAX6* ortholog) mutations cause loss of eye development. The genotypes shown are E15 mouse *Pax6^14Neu/14Neu^* [68], 5 day zebrafish *pax6b^tq253a/tq253a^* [69], human *PAX6^+/^* ^−^ [70], and *Drosophila* ey^−/−^ [71].

Our understanding of gene function is often informed by comparing the phenotypic consequences of mutation with the canonical “wild-type” in a single organism, as well as between mutants of orthologous genes in different organisms. In particular, model organisms have provided great insight into gene function in humans. The importance and need for automating these cross-species comparisons has become imperative as large-scale mutagenesis screens are conducted in model organisms. A fundamental roadblock for analysis is, however, the lack of a computationally tractable method for describing phenotypes that is applicable across multiple domains of biological knowledge and species (for example, see [1]). Not only does each model organism have its own vocabulary for describing the phenotypic consequences of mutation, but these vocabularies are usually tied to the particular anatomies or physiologies of the organism. Often these descriptions are recorded as free text, and although wonderfully expressive, free text remains difficult to reliably compare with computational methods. For example, a computer program would not be able to recognize the fact that there is a significant similarity between the *PAX6* mutations that result in “small eyed” mice, “opaque cornea” in humans, a “malformed retina” in zebrafish, and “eyeless” *Drosophila* (Figure 1).

Current methodologies traditionally identify animal models on the basis of sequence orthology between the mutant animal model and a human gene. For example, Schuhmacher et al. recently developed a mouse model of human Costello syndrome (OMIM: #218040), which is a neuro-cardio-facio-cutaneous developmental syndrome resulting from mutations in the *H-RAS* gene [2]. The mouse *H-Ras* gene was mutated in the orthologous position as in Costello patients, and the resulting phenotype recapitulates the disease. Occasionally, spontaneous models can be identified by the observation of symptoms reminiscent of human disease, for example the *fat aussie* mouse develops obesity, type 2 diabetes, and male infertility. This phenotype is similar to human Alström syndrome, which is caused by mutation in the *ALMS1* gene [3]. Sequencing and further characterization of *fat aussie* revealed a mutation in *Alms1*, and *fat aussie* is emerging as a good animal model for understanding Alström syndrome and the function of cilia-localized *Alms1* [4]. These examples for identifying animal models of disease relied on knowledge of the genetic basis of the human disease, but there are many human diseases for which it is not yet known. If a researcher could compare human model organism, and even ancestral phenotypes directly, they would have a mechanism to more rapidly identify candidate genes and models of disease.

Model organism communities benefit from centralized collections of curated research, where a scientist can search for extensively cross-referenced gene expression, phenotype, and genomic data, referred to as “model organism databases” (MODs). Research in the field of human biology suffers because there is no equivalent resource for the human research community, and linking these diverse datasets requires searching many detached resources. There are, however, several valuable data resources for human phenotypic data, including the *Online Mendelian Inheritance in Man* (OMIM) [5] published by the National Center for Biotechnology Information (NCBI). OMIM contains more than 19,000 records, divided between genes and phenotypes/diseases. Approximately 53% of the gene records have detailed allelic variant descriptions and/or general clinical synopses, while 43% of phenotype/disease records have a known molecular basis. OMIM is a text-based resource, and retrieval of information suffers from this fact, as the Entrez searches in Table 1 show. For an individual researcher wanting to know which human mutations may result in an increase in bone size, or a computer script mining OMIM data, free text annotations do not provide the rigor necessary for querying. While successful mining of the literature to relate genes to phenotypes has been shown [6], it does not provide a mechanism to compare phenotypes directly.

**Table 1:** OMIM query results.

OMIM Query	Number of Records
“large bones”	264
“large bone”	785
“enlarged bones”	87
“enlarged bone”	156
“big bones”	16
“huge bones”	4
“massive bones”	28
“hyperplastic bones”	12
“hyperplastic bone”	40
“bone hyperplasia”	134
“increased bone growth”	612

OMIM text-based query for variants of the phrase “large bones.”

**Figure 2:**
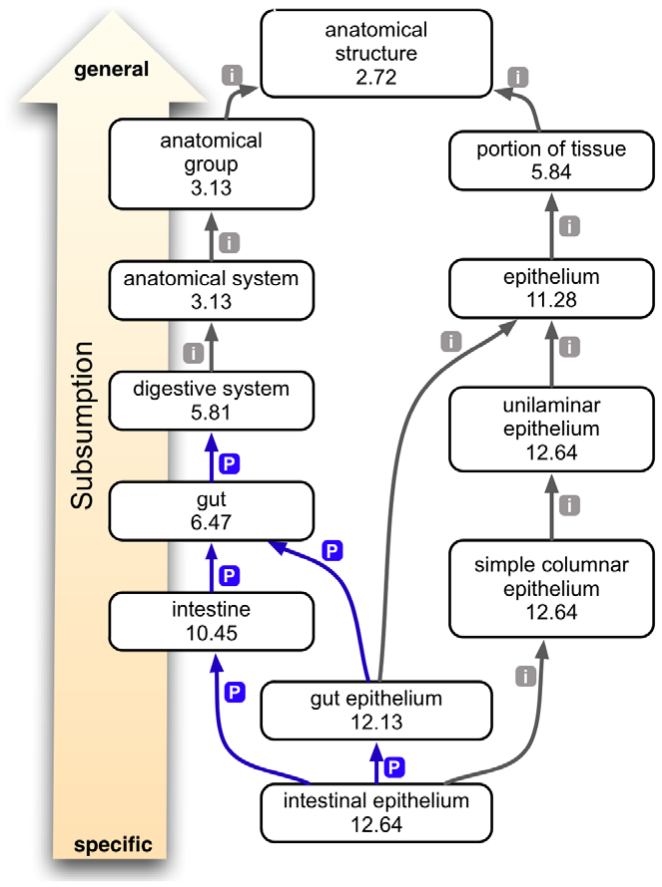
Ontology subsumption reasoning. This example shows the relationships of the term “intestinal epithelium” to other anatomical entities within the ZFA ontology. Gray arrows with an “i” indicate an *is_a* relation, and blue arrows with a “p” indicate a *part_of* relation. The numbers indicate IC of the node, which is the negative log of the probability of that description being used to annotate a gene, allele, or genotype (collectively called a feature). As terms get more general, reading from bottom to top, they have a lower IC score because the more general terms subsume the annotations made to more specific terms.

One of the most revolutionary tools for the biologist has been the ability to compare sequences using algorithms such as BLAST [7], which allows one to quantitatively assess similarity between one or more sequences. However, the genetic basis of a disease is often unknown, and in this case a sequence-comparison tool is of no use to identify sequence mutations. If descriptions of phenotypes were based on a common controlled vocabulary—an *ontology*—they would be structured such that algorithms could be written to compare phenotypes computationally. One of the benefits of using ontologies is the ability to use general-purpose logical inference tools called reasoners (for example, see [8]). Reasoners can assist in query answering and analysis. As an example, consider two different queries, one to find genes expressed in the ZFA:gut, and the other to find genes expressed in the ZFA:epithelium (we write ontology terms prefixed with the name of the ontology; see Materials and Methods for further explanation). We would expect both of these searches to return annotations to the ZFA:intestinal epithelium, because the intestines are a *part_of* the gut, and the intestinal epithelium *is_a* type of epithelium (Figure 2). Analogous to the nucleic and amino acid alphabets and distance matrices used in the BLAST algorithm, ontology terms and their relationships to one another can be used to group and compare phenotypic and gene expression data and can be utilized for cross-species phenotype analysis.

**Figure 3:**
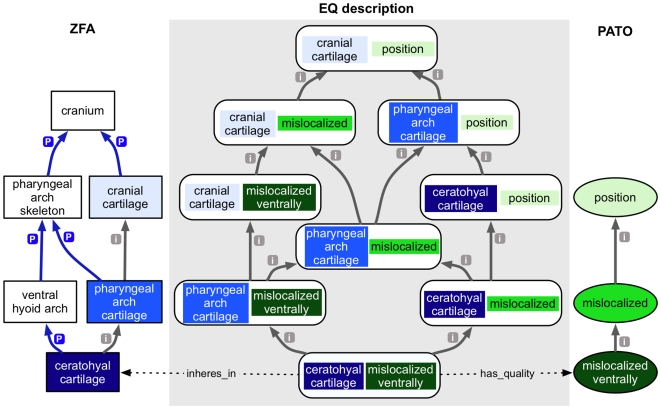
Subsumption reasoning EQ descriptions. The relationship between an EQ description and its contributing ontologies (flanking panels) are shown. The entities are from the ZFA ontology in blue, and the qualities from PATO in green. The full EQ hierarchy (all possible EQ combinations) between ZFA:ceratohyal cartilage + PATO:mislocalized ventrally and ZFA:cranial cartilage + PATO:position are shown, illustrating subsumption across graph nodes comprised of multiple ontology terms. Relationships are as indicated in Figure 2. As with the single ontology in Figure 2, IC scores can be calculated for EQ nodes, where more general EQ nodes having a lower score than more specific EQs.

A phenotype can be defined as the outcome of a given genotype in a particular environment (for review see [9]) and can be described using ontologies to facilitate comparisons. A description of an individual phenotypic character can be recorded using a bipartite “EQ” (Entity + Quality) method, where a bearer entity (such as an anatomical part, cellular process, etc.) is described by a quality (such as small, increased temperature, round, reduced length, etc.). The EQ method is sufficient for the description of many phenotypes, provided the source ontologies are rich enough. The entity terms may be structures from any anatomy ontology, or biological processes, cellular components, or molecular functions from the Gene Ontology (GO) [10]. The quality terms come from the Phenotype and Trait Ontology (PATO), which is designed to be used in combination with species-specific anatomical ontologies or other cross-species entity ontologies (see, for example, [11]–[13]). For instance, a *Drosophila* “redness of eye” phenotype could be described using the terms “red” from PATO and “eye” from the Fly Anatomy ontology (FBbt) into the EQ statement EQ  =  FBbt:eye + PATO:red. The EQ method has been extended to include related qualities and additional entities, and with a post-composition approach to describe more granular entities. Many MODs already utilize community-specific anatomy ontologies, in addition to GO, for annotation of gene expression and/or phenotype data [14],[15], and these methods are described in detail elsewhere [16],[17]. Ontological reasoning can also be applied to EQ descriptions, just as for a single ontology, because they too represent nodes in a graph structure. For example, queries for *cranial cartilage position* should return genotypes that have the phenotype ZFA: ceratohyal + PATO:mislocalised_ventrally. Similarly, queries for superstructures of the ceratohyal cartilage, such as cranial cartilage, should also return these genotypes (Figure 3).

Any EQ description can be combined with other EQ descriptions and data, such as genotype, environment, and stage identifiers from other databases or ontologies, to fully express the phenotypic state of an individual or group. For example, one could record the zebrafish phenotype EQ  =  ZFA:median fin fold + PATO:attenuate at the embryonic stage ZFS:26-somite with genotype *fbn2b^gw1/gw1^*(AB) (defined in the Zebrafish Information Network, ZFIN). With this method, phenotypes can be recorded using multiple ontologies in a highly expressive and finely detailed manner while maintaining correct logic and computability.

Existing computational tools are inadequate to store and analyze this ontology-based phenotype annotation data in a generic, species-neutral way. In particular, there is a lack of tools for the cross-species comparisons needed to identify gene candidates and animal models of disease. Many existing algorithms have been developed and tested using the GO to measure the semantic similarity of annotations and provide a good starting point for analysis (for example, see [18]–[21]). It was unclear how well these algorithms would work for analyzing datasets using a combination of ontologies. Additionally, cross-species comparisons would not be possible because there were no links between the various anatomical ontologies. Schlicker and Albrecht [22] suggest an information content (IC)–based approach to analyzing phenotypic profiles made with multiple ontologies, although they only tested their results with annotations made with the species-neutral GO. Their FunSimMat tool requires a specific list of proteins to compare and therefore does not provide a means to comprehensively search for phenotypically similar genes. PhenomicDB [23] is a cross-species resource that has pulled together annotations from diverse resources and mined free-text phenotypes to provide “phenoclusters” of phenotype-related genes. However, their analysis did not make use of the relationships in the source ontologies. Although known interacting proteins were clustered together, they note that their resulting “phenoclusters” tended to be species-specific due in large part to the community-specific terminologies that were used in the annotations, and not necessarily due to the underlying biology. These existing methods were insufficient for our needs because they were either free-text based or used a limited set of ontologies for annotation, and because they lacked a framework to integrate and compare anatomical entities between organisms. They also lacked metrics for determining significance in similarity calculations. Lastly, apart from the querying aspect, none included a species-neutral method for recording phenotypes de novo.

By annotating phenotypes using this EQ method, together with appropriate computational analysis tools, we have a unique opportunity to standardize and query phenotypic data in a rigorous and illuminating manner. In this study, we tested the hypothesis that EQ annotation of disease phenotypes will facilitate the discovery of new genotype-phenotype relationships within and across species. We EQ-annotated 11 human disease genes from free-text OMIM descriptions with Phenote software [24] to provide a dataset for cross-species comparison. We compared these annotations to annotations of the mouse and zebrafish orthologs, which required the development of a cross-species unifying ontology (UBERON) to provide a bridge between different anatomy ontologies. We also developed new, and extended existing, metrics for measuring the phenotypic similarity between genes. We assessed their relative performance through analysis of known signaling pathways and genetic interactions and show that these data can be queried and compared by phenotype *alone* to identify biologically meaningful similarities. Furthermore, these annotations provide a resource for a better understanding of existing disease phenotypes. We conclude that this method can facilitate the discovery of new genotype-phenotype associations within and between species.

## Results

### Selection, Annotation, and Analysis of OMIM Genes

Although many MODs curate phenotype data using the EQ method, no such annotations existed for human disease genes. Because we required annotations of human diseases in the EQ style for comparison, we proceeded to annotate a small set of gene records from OMIM: *ATP2A1*, *EPB41*, *EXT2*, *EYA1*, *FECH*, *PAX2*, *SHH*, *SOX9*, *SOX10*, *TTN*, and *TNNT2*. These 11 genes were selected because they were known to be causal for a variety of human diseases and had known mutant orthologs in flies, mice, and/or fish with corresponding EQ descriptions available for comparative analysis.

Specifically, our curation process involved translating OMIM textual descriptions into associations between genotypes and phenotypes, where the phenotypes were delineated using EQ descriptions. Specific ontologies were chosen based on their community-wide acceptance and use, as well as their species-specificity and granularity. For annotation of human disease genes from OMIM, and their resulting phenotypes, we utilized the Foundational Model of Anatomy for adult human gross anatomy (FMA [25]) and the human developmental anatomy ontology (EHDAA) for developing anatomical structures. Additionally we utilized the cell ontology for cell types (CL [26]), CHEBI for chemicals [27], the GO for sub-cellular components and biological processes, and PATO as the source of qualities presented by these varied entities.

Free-text phenotype or disease description was translated into one or more individual EQ phenotypic descriptions, so that a single genotype (i.e., one or more variant alleles plus the genetic background, to whatever extent it is known) could be associated with multiple EQ descriptions. In the following sections, we refer to a “phenotypic profile” as the sum-total of the EQ descriptions for an individual genotype. For example, Figure 1 shows phenotypic profiles for eye phenotypes of *PAX6* ortholog mutations in mouse, human, zebrafish, and fruitfly (also see Table 2). An important thing to note is that any given individual organism presenting a phenotype may manifest only a subset of the EQ descriptions of a complete phenotypic profile for a particular genotype. The *PAX6* and ortholog EQ descriptions are based on gross observations of individual eyes, at a particular developmental stage. These genotypes have additional phenotypes not shown in Figure 1 (different anatomical structures, at other developmental stages, and so forth) that would contribute to their complete phenotypic profile. Alternatively, other *PAX6* genotypes may have different (or similar) phenotypic profiles. Therefore, the phenotypic profile for each genotype grows with time as more observations are made, and this information is easily associated with the allele or gene level for comparison.

**Table 2:** Free-text to phenotypic profile extraction example.

EQ Descriptions	
Entity	Quality
GO:sensory perception of sound	PATO:disrupted
FMA:External_ear	PATO:structure
FMA:Middle_ear	PATO:structure
FMA:internal_ear	PATO:structure
EHDAA:branchial_arch	PATO:structure, cavities
EHDAA:branchial_arch	PATO: cystic
FMA:Kidney	PATO:decreased size
GO:kidney_development	PATO:arrested
FMA:Kidney	PATO:absent
GO:sensory perception of sound	PATO:disrupted

The following free-text describing the branchiootorenal syndrome I (OMIM#113650) phenotype is annotated using multiple EQ phenotype descriptions: “sensorineural, conductive, or mixed hearing loss, structural defects of the outer, middle, and inner ear, branchial fistulas or cysts, and renal abnormalities ranging from mild hypoplasia to complete absence.” EHDAA, Human Developmental Anatomy; FMA, Foundational Model of Anatomy; GO, Gene Ontology; PATO, quality ontology.

For the 11 selected human disease genes, curators annotated the general description of the phenotypes contained within the body of each OMIM gene record to a general OMIM gene identifier (i.e., OMIM:601653). Additionally, any mention of specific alleles was curated to the allelic variant ID (i.e., OMIM:601653.0001). Therefore, the general OMIM ID is representative of all non-indicated alleles, rather than a general phenotype description of all alleles. Five of the 11 genes were recorded independently by three curators to test for annotation consistency (to be published elsewhere). In total, 1,848 annotations comprising 709 distinct descriptions were collected for all 11 genes with 114 alleles (Table 3). Some descriptions were frequently identical, such as the description EQ  =  FMA:palate + PATO:cleft being used to annotate 25 genotypes of 3 genes. Of these 709 descriptions, 487 used FMA, 110 used GO, and 4 used CL ontologies to describe the entities.

**Table 3:** Phenotype profile statistics for EQ-annotated OMIM genes.

Gene	Number of Genotypes	Number of Annotations	Distinct EQs
ATP2A1	5	16	3
EPB41	5	18	8
EXT2	5	35	7
**EYA1**	20	567	137
FECH	14	37	9
**PAX2**	17	178	87
SHH	23	215	31
**SOX9**	14	329	164
**SOX10**	19	298	155
TNNT2	10	36	7
**TTN**	27	143	59

For each OMIM gene, the number of alleles annotated and the total number of EQ annotations are listed. Of these total number of annotations, the number of which were unique amongst the set are also listed. This set of annotations provides the basis for the analysis presented in Figure 5. Genes annotated in triplicate are indicated in **bold**.

### Comparative Analysis between Phenotypic Profiles

We loaded all annotations and source ontologies (Table 4) into a single OBD instance [28]. Briefly, this is an information system that allows for the construction of complex descriptions using multiple ontologies, and logical reasoning over these descriptions and the annotations that utilize them. OBD also has analysis capabilities that support comparison of like entities (such as genes, alleles, and genotypes) based on their shared attributes (such as their phenotype profiles). The reasoning step is required for the comparison step.

**Table 4:** Annotation Sources.

Source	Ontologies Used	Number of Genes	Number of Unique Descriptions
OMIM^1^	EDHAA, FMA, GO, SO, ChEBI, PATO	11	709
MGI^2^	MP, GO, PATO	10,579	5,266
ZFIN^3^	ZFA, ZFS, GO, PATO	2,911	5,157
GAD^4^	MP, DO	2,674	1,792

Data were comprised of annotations and ontologies from a variety of sources. Listed are the ontologies used in the annotations from each data source, along with the number of genes annotated, and the number of unique phenotype descriptions (EQs). Annotations from MGI and GAD were made using the pre-coordinated ontologies MP and DO.

1 this study.

2 http://mgi.org.

3 http://www.zfin.org.

4 http://www.gad.org.

OBD assigns an IC score to every term or EQ description used to annotate a gene, allele, or genotype. The IC score is a measure of how informative a term or a description is, based on the frequency of annotations with the term and depth in the ontology. The IC score will thus vary depending on the background set of annotations. OBD uses a reasoner to compute IC scores, such that annotations “propagate up the graph,” and consequently more general terms receive lower IC scores. For example, Figure 2 shows nodes from the zebrafish anatomy (ZFA) ontology, each with an IC score. Terms deeper in the ontology are more distinguishing and informative (i.e., a term such as ZFA:intestinal epithelium has a higher score, IC  = 12.4) than those at the root (i.e., ZFA:anatomical structure, IC  = 2.72), because all intestinal epithelium phenotypes are also anatomical structure phenotypes. OBD treats phenotypic EQ descriptions in the same way as other terms, and these nodes are assigned IC scores in the same fashion. Just as for the terms, the reasoner can calculate annotation frequencies such that more general EQ descriptions such as ZFA:cranial cartilage + PATO:position have lower IC scores than more specific, less frequently used, and thus more informative descriptions such as ZFA:ceratohyal cartilage + PATO:misplaced ventrally (Figure 3).

**Figure 4:**
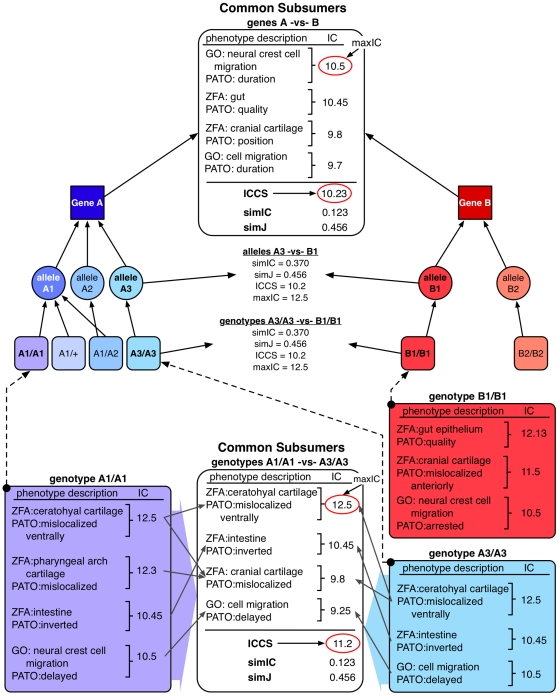
Phenotypic profile comparison and phenotype promotion. Multiple EQ descriptions annotated to a genotype comprise a phenotypic profile, and these profiles can be compared using subsumption logic. Phenotypes annotated to genotypes are propagated to their allele(s), and in turn to the gene, indicated with upward arrows. Similarity is analyzed between any two nodes of the same type, such as between gene A-vs-B, allele A3-vs-B1, genotypes A1/A1-vs-A3/A3, or A3/A3-vs-B1/B1. Genotypes are shown as rounded boxes, alleles as circles, and genes as squares. The phenotypic profile of genotype A1/A1 is detailed in purple, genotype A3/A3 in blue, and B1/B1 in red. The common subsuming phenotypes between A1/A1-vs-A3/A3 and gene A-vs-B are itemized in white boxes. Arrows between the original phenotypic descriptions and their common subsuming phenotypic description are indicated. Some individual phenotypic descriptions can have two common subsumers. For each phenotypic description (EQ), the calculated IC is shown. When comparing two items, four scores are determined: maxIC, the maximum IC score for the common subsuming EQ, which may be a direct (in the case of A1/A1-vs-A3/A3) or inferred (in the case of gene A-vs-gene B) phenotype, circled in red; avgICCS, the average of all common subsuming IC scores; simIC, the similarity score which computes the ratio of the sum of IC values for EQ descriptions (including subsuming descriptions) held in common (intersection) to that of the total set (union); simJ, non-IC-based similarity score calculated with the Jaccard algorithm which is the ratio of the count of all nodes in common to nodes not in common. These scores are also indicated for the comparisons between alleles A3-vs-B1 and A3/A3-vs-B1/B1, although the full profile is not being shown.

OBD can utilize the IC scores of each node to compute various measures of similarity between any two pairs of annotations or phenotypic profiles. We utilized three IC-based metrics as calculated in OBD to perform our analysis in this paper: similarity based on Information Content (simIC), Information Content of the Common Subsumer (ICCS), and maximal Information Content of a pair (maxIC). A non-IC-based metric, the Jaccard similarity coefficient (simJ), was also included in our analysis. These metrics are detailed in [28] and [18] and in the Materials and Methods section below. Figure 4 shows an example of how these different metrics result from a set of genotypes being compared and how phenotypic profiles are promoted to the alleles and genes for comparison at those levels. The simIC metric quantifies the similarity between two phenotypic profiles using the reasoner to determine which EQ phenotype descriptions are shared based on the subsumption hierarchy. If two phenotypic profiles are very similar, we expect their profiles to converge more quickly and share quite specific phenotype descriptions (i.e., with high IC scores); conversely, dissimilar profiles will share only a few very general phenotype descriptions in common (i.e., with low IC scores). Each subsuming EQ also has an IC, and the average of the resulting set of the EQs in common provides the ICCS score. Of this set of EQs that subsume two phenotypic profiles, one will have the highest IC, the maxIC of all pairs. The simJ metric does not use IC but is rather a ratio of the count of all nodes in common to nodes not in common based on the hierarchy.

We can directly compare any two items of the same type, such as two genotypes, two alleles, or two genes by promoting annotations from the genotype carrying a particular allele up to the allele itself, or to the affected gene. Figure 4 illustrates the comparison of two phenotypic profiles at the genotype and gene levels, and the calculation of similarity metrics at those different levels. The two profiles share a total of four common subsumers; some of the annotations have a single common subsumer of the different genotypes; others map to two different common subsumers. In this example, genotypes A1/A1 and A3/A3 share an identical annotation to ZFA:ceratohyal cartilage + PATO:mislocalized ventrally with an IC  = 12.5, which is therefore one of the common subsuming annotations and, in this case, also the highest scoring common subsumer, or maxIC. As detailed in Figure 3, ZFA:ceratohyal cartilage + PATO:mislocalized ventrally and ZFA:pharyngeal arch cartilage + PATO:mislocalized phenotypes share the common subsuming parent ZFA:cranial cartilage + PATO:mislocalized. Therefore, the common phenotypes that subsume genotypes A1/A1 and A3/A3 include both of these parent EQ descriptions. The phenotypes of A1/A1 and A3/A3 are promoted up to the alleles A1 and A3, respectively, and in turn to gene A. In this example, when the comparison is made at the gene level, the highest scoring common subsumer (the phenotype with the maxIC) is GO:neural crest cell migration + PATO:duration. The common subsumers of annotations to the anatomy terms are at more generic nodes, due to their convergence point in the ontologies (Figures 2 and 3).

### Phenotype Comparison between Allelic Variants

The first test to assess how well the EQ annotation and phenotype comparison methods work was to correctly identify alleles of the same gene based on their phenotype descriptions. We compared the phenotypic profiles of all pair-wise combinations of alleles annotated for each of the 11 OMIM genes using four scoring metrics in OBD (simIC, ICCS, simJ, and maxIC). Our hypothesis was that similarity scores between alleles of the same gene (i.e., intra-gene) would be significantly higher than similarity scores between either one of these alleles and alleles of other genes (i.e., inter-gene). Only monogenic phenotypic profiles were included in this part of our analysis; digenic genotypes were not included (for example, OMIM:600725.0011/OMIM:603073 has a double mutation in *SHH* and *ZIC2*).

**Figure 5:**
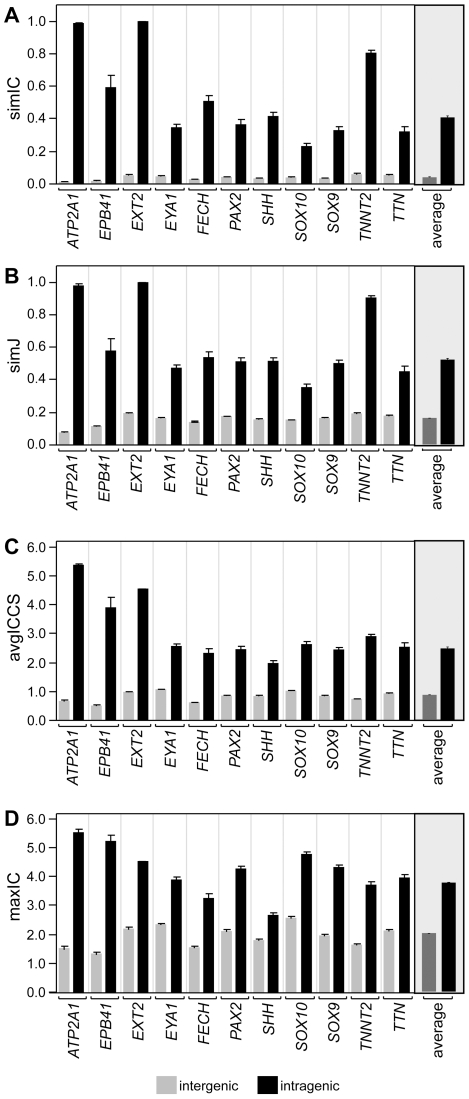
Similarity metrics analysis of phenotype profiles between and within genes. Each of the four panels shows one of the four similarity measurements, comparing the score for alleles of the same gene (intra, in black) versus alleles of all other genes (inter, in gray), for each of the 11 OMIM genes annotated. The average of all 11 OMIM gene comparisons for each similarity metric are shown in the grayed portion of the graph on the right. Metrics are (as described in Figure 4): (A) simIC, (B) simJ, (C) ICCS, and (D) maxIC. For each metric, there was a significantly higher similarity value (*p*<0.0001) for the intra-genic comparisons as compared to the inter-genic comparisons. Significance was tested using a two-tailed Student's *t*-test, for the pairwise comparison (intra versus inter) for all four metrics for each gene. Error bars are standard error of the mean.

Figure 5 summarizes the results, showing that without exception, intra-gene allelic variants were more phenotypically similar (*p*<0.0001 in two-tailed *t*-test) to each other than to those of other genes using any of the four metrics. Another way to examine the similarity between genetic variants is to use each allele to query all other alleles to determine which other allele is most similar. Out of all 118 alleles in the analysis, all had their most phenotypically similar genotype in the same gene. Together, these results support our hypothesis that EQ-based phenotype descriptions capture the similarities between alleles of the same gene, and these ontology-based similarity metrics are effective in retrieving related alleles and quantifying their phenotypic similarity.

### Retrieval of Pathway Genes by Phenotype Query

Members of a signaling pathway frequently exhibit similar mutant phenotypes, and therefore we predicted that a query based on the phenotype due to a mutation in one member of a pathway would retrieve other known members of that pathway. We tested this hypothesis on the well-characterized hedgehog-signaling pathway, which regulates patterning and midline development in animals [29]. ZFIN has >2,900 genes with mutant phenotypes annotated with the EQ method [13], including 20 of the 64 known hedgehog pathway members identified in ZFIN [30]. The entity terms were typically drawn from the zebrafish-specific anatomical ontology, as well as from GO, and the quality terms were from PATO. The annotations from ZFIN (17,494 total, 5,157 unique descriptions) were loaded together with the source ontologies (Table 4).

**Figure 6:**
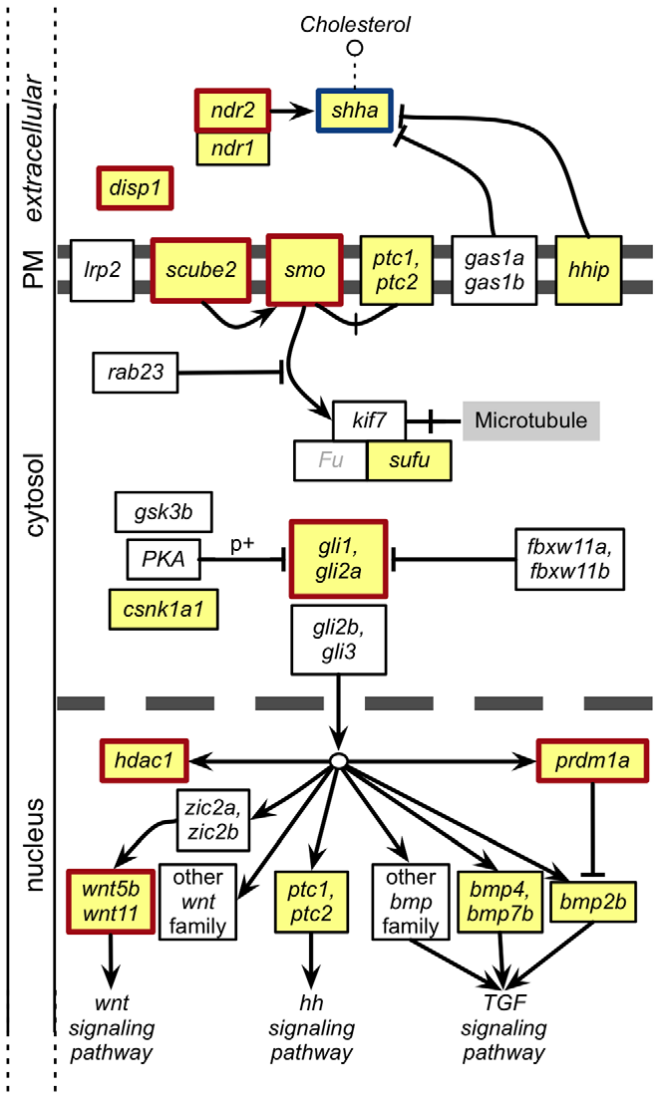
A similarity search for mutant phenotypes similar to zebrafish *shha* retrieves many known pathway members. Based on the diagram from KEGG [31], the double gray line represents the plasma membrane, and the dashed line the nuclear membrane. All known shha pathway members are shown; those with recorded mutant EQ annotations are yellow. Pathway members retrieved in the top 23 most similar genes are indicated by red boxes. Known pathway members in ZFIN are shown with their current nomenclature, with the exception of those with uninformative nomenclature, which are listed with their KEGG reference gene family nomenclature and are capitalized. KEGG reference pathway members not yet identified in zebrafish (Fu) are grayed out.

We queried OBD for genes with mutant phenotypes similar to the mutant phenotype of the zebrafish *shha* gene (ZDB-GENE-980526-166). Figure 6 illustrates these results based on the zebrafish hedgehog signaling pathway diagram from KEGG [31], to which some additional genes have been added based on current knowledge [30]. Table 5 lists the hedgehog pathway members, and other phenotypes significantly similar to *shha*, in order of their rank by simIC, together with their ranks and scores by the four metrics. Six of the 11 genes scoring as most similar by simIC are known to be members of the hedgehog signaling pathway, seven by simJ, five by ICCS, and three of the top eight by maxIC (many genes were tied for ninth place, see Table S1). This set of the most similar genes to *shha* comprised 23 genes total, of which 11 were known pathway members. Assuming a hypergeometric distribution, the chances of retrieving 11 of the 20 mutant pathway members in the top 23 out of 2,908 genes at random is very low (*p*<E-19). Three known pathway members, *bmp2b*, *hhip*, and *sufu*, were not identified in the top 10 most similar. *sufu* was the lowest ranking of these at 628 of the 2,908 genes compared by simIC (see Table S1 for additional metrics). To further test the similarity algorithm, we performed the reverse query to determine if any hedgehog pathway members were similar to *sufu*. The most similar pathway member to *sufu* was *hhip* (rank 3 by simIC).

**Table 5:** Zebrafish genes with similar phenotypes to zebrafish *shha*.

Gene	simIC	simJ	ICCS	maxIC	Role in Hedgehog Pathway	Ref
***disp1*** ^a^	**1**	**1**	38	43	Regulates secretion of lipid modified *shh* from midline.	[72]
***gli2a*** ^a^	**2**	**3**	**1**	**1**	Zinc finger transcription factor target of *shh* signaling.	[73]
***lama1***	**3**	**2**	35	127	Basement membrane protein important for eye/body axis development.	[74]
***smo*** ^a^	**4**	**4**	**2**	**1**	Membrane protein binds *shh* receptor *ptc1*.	[75]
***scube2*** ^a^	**5**	18	118	43	May act during *shh* signal transduction at the plasma membrane.	[76]
***prdm1a*** ^a^	**6**	**10**	31	43	Zinc-finger domain transcription factor, downstream target of *shh* signaling.	[77]
***dharma***	**7**	**5**	56	57	Paired type homeodomain protein that has dorsal organizer inducing activity and is regulated by *wnt* signaling.	[78]
***gli1*** ^a^	**8**	**6**	21	57	Zinc finger transcription factor target of *shh* signaling.	[79]
***extl3***	**9**	**7**	75	127	Glycosyltransferase involved in heparan sulfate biosynthesis, required for optic tract sorting by *robo2*.	[80]
***ext2***	**10**	11	133	127	Glycosyltransferase involved in heparan sulfate biosynthesis, required for limb development.	[81],[82]
***hdac1*** ^a^	11	**8**	**4**	**7**	Transcriptional regulator required for *shh* mediated expression of *olig2* in ventral hindbrain.	[83]
***ndr2*** ^a^	14	**9**	36	57	TGFbeta family member upstream of hedgehog signaling in the ventral neural tube (aka cyclops).	[84]
***kny***	15	14	**6**	9	Glypican component of the *wnt*/PCP pathway.	[85]
*doc*	16	48	94	43	Unmapped; identified in large-scale screen with several other pathway members; affects notochord, somite formation, and patterning.	[40]
***vangl2***	20	17	**5**	9	Modulates *wnt*/PCP signaling pathway during gastrulation.	[36]
***wnt11*** ^a^	22	21	**8**	32	Extracellular cysteine rich glycoprotein required for *gli2/3* induced mesoderm development.	[86]
***wnt5b*** ^a^	29	33	**3**	32	Extracellular cysteine rich glycoprotein required for convergent extension movements during posterior segmentation.	[87]
***robo2***	44	50	17	**1**	Signals olfactory axon guidance along midline in forebrain (*Shh* acts as axon guidance ligand through *robo*-related proteins *Boc/Cdon* in mouse).	[88],[89]
***cho***	50	81	**7**	9	Unmapped; identified in large-scale screen with several other pathway members; affects somite patterning and pigment cells.	[40]
*bmp2b* ^a^	71	72	62	103	Downstream target of *gli2* gene repression.	[90]
***chd***	78	44	16	**1**	Negative regulator of *bmp* signaling, normally coexpressed with *shh* in notochord (chick).	[91],[92]
***tbx24***	141	395	**10**	**7**	A T-box transcription factor expressed in presomitic mesoderm (PSM) and involved in PSM maturation, independent of *Notch*.	[93]
*ptc2* ^a^	154	102	24	43	Membrane receptor for *shh*.	[94]
***cdh2***	171	126	**9**	21	A cell adhesion molecule expressed in the neural tube and required for neural tube closure, regulated by *ndr1/2*.	[95]
*ptc1* ^a^	188	140	33	43	Membrane receptor for *shh*.	[96]
***chaf1b***	194	148	25	**1**	A chromatin assembly factor that requires *shh* and *hdac1* activity—required for cell cycle exit and differentiation in zf retina.	[97]
***plxna3***	212	285	22	**1**	A membrane protein, semaphorin receptor, which regulates intraspinal motor axon guidance (*shh* known to act as axon guidance ligand).	[98],[99]
*ndr1* ^a^	224	262	20	9	TGFbeta family member upstream of hedgehog signaling in the ventral neural tube (aka “squint”).	[95]
*hhip* ^a^	325	300	262	321	Binds *shh* in membrane and modulates interaction with *smo*.	[75]
*sufu* ^a^	628	553	257	395	Signal transduction of *hh* signal.	[100]

All genes ranking in the top 23 are listed, ordered by rank of simIC, together with their ranks by all metrics and a short description of a putative function of the gene product with particular reference to the hedgehog pathway. Known hedgehog pathway members indicated with an ^a^. The rank for each score (simIC, simJ, ICCS, and maxIC) was determined by its position in a sorted list, with tied rankings representing a shared score; next-lower scores ranked at their position in the list. The set of the top 23 most similar genes includes the top 10 by each metric, with the exception of maxIC, where the top eight were included due to many ties. The chances of retrieving 11 of the 20 pathway members, based on a hypergeometric distribution, in the top 23 out of 2,908 genes is very low (*p*<E-19). Genes that scored in the top 23 are in **bold**. A full table of results is listed in Table S1.

Intriguing are the additional zebrafish mutants found to have highly similar phenotypes (for example, *lama1*, *dharma*, *ntl*, and *doc*), but which are as yet unlinked to the hedgehog pathway, either because they are not yet mapped or are untested in this role. These results show that known, and potentially new, pathway members within the same species can be identified using EQ methodology and the similarity algorithms available within OBD.

### Comparing Phenotypes of Cross-Species Orthologs

**Figure 7:**
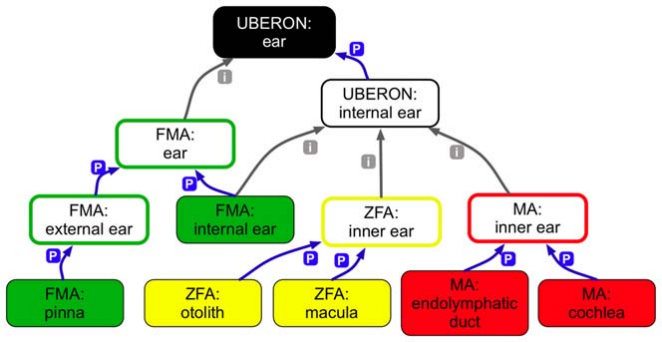
UBERON links multiple species-specific anatomy ontologies. The entities for selected human, zebrafish, and mouse *EYA1* phenotypes were annotated using species-specific anatomy ontologies (FMA, ZFA, and MA, respectively) as indicated by the solid squares. Outlined squares indicate entities of subsuming annotations, color coded to match the source ontology. Annotations can be associated with common subsuming nodes via UBERON. In this example, each of the annotated entities can be linked through the UBERON:ear (black).

One of the primary goals of this study was to compare phenotypes across species directly, particularly human to model systems. This goal presented two challenges; first, we needed to include more annotations from additional sources, specifically mouse annotations from MGI [32],[33], and disease associations from the human Gene Association Database (GAD) [34]. However, these annotations were described using neither PATO nor an anatomical ontology. The MGI annotations use the Mammalian Phenotype (MP) ontology, and the GAD uses textual descriptors. To integrate these valuable data, we first created an equivalence mapping of MP terms to EQ descriptions [17]. We also mapped the GAD descriptors to Disease Ontology (DO) terms and created a mapping of DO terms to the FMA. These annotations, together with their source ontologies, were loaded into OBD (Table 4). The second challenge in making cross-species comparisons is that each species of interest has its own unique anatomical ontology. This means that there is no automated method to determine that a zebrafish ZFA:cranial nerve VII phenotype is in fact related to a human FMA:facial nerve phenotype. In initial tests, orthologs scored very poorly in terms of phenotypic profile matches, as might be expected (unpublished data). The majority (85%) of annotations in OBD were made using these species-specific anatomical ontologies, and without a means for linking them across species, only species-neutral ontologies such as GO, CL, and PATO could be used for comparisons. We recognized that the comparisons would be greatly enhanced by providing links between the anatomical structures in the different organismal anatomy ontologies that would allow the search algorithm to identify commonalities in the phenotypic profiles of different organisms. Therefore, we added UBERON to OBD, a multi-species ontology which generalizes over the types of structures represented in the species-centric anatomical ontologies and provides links between these terms and UBERON terms (see Methods) [16]. For example, Figure 7 shows how phenotype annotations to the mouse MA:cochlea, the zebrafish ZFA:macula, and the human FMA:pinna may be related via the common superclass ear in UBERON.

Our final hypothesis was that sequence orthologs would exhibit similar mutant phenotypes and therefore phenotype descriptions alone would be sufficient to identify orthologs and pathway members. To test this, we queried the complete set of zebrafish and mouse phenotypes, using the phenotypic profiles of the 11 human disease genes annotated from OMIM and our four scoring metrics. Table 6 shows the score and rank of the mouse and zebrafish orthologs when compared to the human disease gene for all four metrics. The full set of returned genes for zebrafish and mouse using all four metrics are available in Table S2–S23. In the case of the human-zebrafish comparison, seven out of the 11 orthologous genes were returned in the most similar 100 by any metric, with five being in the top 10 by two or more metrics. Three zebrafish genes, *pax2a*, *sox10*, and *ttna*, were found to be the most similar to their human ortholog (rank 1 by ICCS and maxIC metrics, as well as by simIC for *sox10*). The human-mouse comparison revealed fewer orthologous findings, with only 5 of the 10 orthologs (no annotations for mouse *Tnnt2* were available at the time of analysis) being identified in the most similar 100 genes by any of the metrics. Of these five, four were in the top 10 by two or more metrics. Two mouse genes, *Ebp4.1* and *Eya1*, were the most similar to the human ortholog by two metrics. In some cases, the rankings of the orthologous gene were very similar by the different metrics. For example, comparison of human and mouse *EPB41* ranked the mouse ortholog first in the case of ICCS and maxIC, sixth for the simJ metric, and third for the simIC metric. In other cases, the rankings were more variable for the different metrics. For example, mouse *Pax2* was ranked as only 45th by the simJ metric, but in the top 10 most similar genes by the simIC and ICCS metrics.

**Table 6:** Ortholog rankings of phenotype similarity search using human disease genes.

		Mouse				Zebrafish			
		simIC	simJ	ICCS	maxIC	simIC	simJ	ICCS	maxIC
***ATP2A1***	**rank**	NF	NF	NF	NF	NF	NF	NF	NF
	score	0.005	0.054	0.844	1.52	0.025	0.086	1.99	4.2
***EPB41***	**rank**	**3**	**6**	**1**	**1**	180	130	185	134
	score	**0.09**	**0.197**	**5.39**	**10.41**	0.017	0.121	1.55	2.88
***EXT2***	**rank**	NF	NF	NF	NF	NF	NF	NF	NF
	score	0.017	0.101	2.08	3.63	0.009	0.07	1.29	2.71
***EYA1***	**rank**	**1**	**1**	**5**	26	**4**	**5**	**2**	22
	score	**0.075**	**0.159**	**5.43**	10.56	**0.029**	**0.085**	**4.4**	10.27
***FECH***	**rank**	NF	NF	NF	NF	NF	NF	NF	NF
	score	0.034	0.119	3.14	9.83	0.005	0.066	0.63	1.67
***PAX2***	**rank**	**7**	45	**3**	31	**9**	16	**1**	**1**
	score	**0.077**	0.168	**4.83**	9.09	**0.039**	0.096	**4.6**	**12.73**
***SHH***	**rank**	NF	NF	NF	NF	15	16	63	18
	score	0.062	0.116	4.69	10.93	0.04	0.119	3.26	6.89
***SOX9***	**rank**	**3**	**2**	**4**	11	**7**	**8**	**2**	**2**
	score	**0.066**	**0.132**	**5.07**	11.15	**0.025**	**0.079**	**4.08**	**12.15**
***SOX10***	**rank**	NF	NF	NF	NF	**1**	**2**	**1**	**1**
	score	0.098	0.077	4.23	9.62	**0.06**	**0.126**	**5.38**	**12.73**
***TNNT2***	**rank**	—	—	—	—	117	210	161	**22**
	score	—	—	—	—	0.018	0.093	4.66	**2.16**
***TTN***	**rank**	23	31	35	**6**	**2**	**2**	**1**	**1**
	score	0.05	0.131	4.31	**10.73**	**0.038**	**0.116**	**4.92**	**12.73**

The four similarity metrics are reported for each human-mouse or human-zebrafish ortholog pair. The rank shows where the ortholog is returned using each similarity metric in the top 250 most similar genes (by simJ) with the human gene queried against all mouse or zebrafish genes, respectively. “NF” indicates that the ortholog was not found in the top 250 genes. Cases where the orthologs ranked in the top 10 are **bold**. No comparison between human *TNNT2* and mouse *Tnnt2* could be made, because no mouse annotations were available at the time that OBD was loaded. In cases where two zebrafish paralogs existed, the “a” gene was used for comparison: *pax2a*, *shha*, and *sox9a*.

Because the most phenotypically similar gene by the four metrics was often not the sequence ortholog, we took a closer look at which genes were the most similar. Table 7 lists the mouse and zebrafish genes most phenotypically similar (rank 1) to the 11 human disease genes, for each of the four metrics. In general, there did not appear to be a significant bias towards one metric in the first-place ranking of orthologs. One ortholog was returned as most similar by each metric in the mouse, and one by simIC, and three each by maxIC and ICCS in the zebrafish. Some of the most similar genes are in the same family as the ortholog (for example, mouse *Epb4.1*, *Epb4.2*, and *Epb4.9*; and zebrafish *sox9a* and *sox10*). Other similar genes may participate in the same pathway, for example, mouse *Shh* and *Cdon*. Some of the returned genes are known to function in similar locations, such as *atp2a1* and *ryr1b*, which are both sarcoplasmic reticulum calcium channels. These results show that the EQ method of describing phenotypes with species-specific ontologies (FMA, ZFA, and MP), when combined with species-neutral ontologies (PATO, GO, CL, and ChEBI) and a species-neutral linking ontology (UBERON), can be used to successfully query for similar phenotypes across species using the similarity algorithms available in OBD.

**Table 7:** Genes most phenotypically similar to human disease genes.

	Mouse				Zebrafish			
Gene	simIC	simJ	ICCS	maxIC	simIC	simJ	ICCS	maxIC
*ATP2A1*	*Jph1*	*Slc25a5*	*Aldh2, Cisd1*	*Jph1*	*ryr1b*	*ryr1b*	*ryr1b*	*ryr1b*
*EPB41*	*Epb4.9*	*Mnek1a*	***Epb4.1***	***Epb4.1*** *, Epb4.2, Epb4.9, Trf*	*smad5*	*gata1*	*dtl*	*dtl, kiaa1279, sass6, stil*
*EXT2*	*Hoxd8*	*Hoxd8*	*Hoxc4*	*Sp7, Crtap*	*unm t30212*	*unm t30539*	*unm t30611, unm t30441, unm t30362, unm t30361, unm t30442, unm t30604, unm t30748*	*dla, blo, exp, stb, unm tz227c, unm tg310a*
*EYA1*	***Eya1***	***Eya1***	*Tbx1*	*Trps1, Gja1, Msx2*	*rerea*	*fgf8a*	*rerea*	*axin1, chm, shy*
*FECH*	*Abcg2*	*Abcg2*	*Abcg2*	*Anapc2, Usp8*	*tal1*	*abhd11*	*kita*	*tal1*
*PAX2*	*Rpl24*	*Maf*	*Mitf*	*Mitf*	*lamb1*	*sufu*	***pax2a***	***pax2a*** *, flr, axin1*
*SHH*	*Cdon*	*Ctnnbip1*	*Alx1*	*Ift57*	*rerea*	*fgf8a*	*sox9a*	*sox9a, tfap2a,*
*SOX9*	*Fgfr2*	*Ror2*	*Prrx1*	*Ror2, Fgfr3*	*fgf8a*	*cdc16*	*fgf8a*	*int*
*SOX10*	*Ednrb*	*Ednrb*	*Ednrb*	*Ret*	***sox10***	*mib*	***sox10***	***sox10*** *, pbx4, ache, tfap2a, tcf7l2, psoriasis*
*TNNT2*	*Hdac9*	*Hdac9*	*Irx4*	*Hdac9*+20 tied	*cx36.7*	*cx36.7*	*vmhc*	*acvr1,ttna*
*TTN*	*Myl2*	*Scn5a*	*Mybpc3*	*Myl2, Nkx2–5*	*cx36.7*	*cx36.7*	***ttna***	***ttna*** *, mef2ca, ache, hey2*

Shown are the highest scoring genes when comparing a human gene versus either mouse or zebrafish, using the four different similarity metrics (as in Table 6). Sequence orthologs that are the top hit are in **bold**. For maxIC, there were often ties for the top rank, which are all listed with the exception of *Tnnt2* versus mouse, where there were 20 genes ranked as the most similar. Please see Tables S2–S23 for a full listing.

## Discussion

### Assessing the Method

This is the first effort to systematically record, and computationally compare, phenotype descriptions with the goal of providing a new tool for discovering genotype-phenotype relationships within and across species. We tested our methods incrementally, showing: first, that allelic variants were most phenotypically similar to other allelic variants of the same gene; second, that we could retrieve known pathway members based on the similarity of the mutant phenotypes; and third, that we could identify orthologous genes across species. Together, these tests indicate that automated similarity analysis of structured phenotype descriptions can successfully identify sets of genes with important and informative biological relevance. Specifically, EQ phenotype description used in combination with IC-based similarity metrics and anatomical mapping between organisms provides the resources necessary for both precisely recording the phenotypes observed and subsequent computational comparisons, which are unconstrained by terminological differences between research communities.

#### Phenotypic similarity of alleles

In applying the phenotypic similarity metrics to our data, we first compared the alleles of 11 human genes and found that the four metrics (simJ, simIC, ICCS, and maxIC) all ranked other alleles of the same gene as the most similar (Figure 5). On average, alleles of the same gene scored 2-fold more similar than alleles of different genes. These results suggested that EQ-based phenotype descriptions, and the similarity scores computed based on these descriptions, were sufficient to retrieve related alleles and measure their relative phenotypic similarity.

#### Phenotypic similarity of signaling pathway members

Our second test was to determine whether we could retrieve other known pathway members based on their having similar mutant phenotypes. A query using the zebrafish *shha* gene returned 16 of 20 EQ annotated known pathway members in the top 10% by all metrics (select additional genes shown in Table 5, all results in Table S1). Furthermore, in a combined list of the 23 most similar genes by all metrics, 11 of the 20 annotated mutant pathway members were identified. The chances of retrieving these randomly are exceedingly low. Furthermore, based on current literature, the additional genes that were found show strong potential for playing a role in the hedgehog pathway and provide interesting candidates for further study.

For example, *lama1*, a Laminin essential for normal lens development, scores as highly similar by simIC (rank 3). At the time of this writing, *lama1* was not yet linked specifically to *shha* in zebrafish. However, it has since been shown that mouse *Shh* directly binds to *Laminin* and that the *Shh-* *Laminin* complex induces cell proliferation in granule cell precursors in the external germinal layer during CNS development [35]. Recently, *lama1* has also been shown to interact genetically with *vangl2* in zebrafish, another gene found to score as highly similar to *shha* (rank 3 by maxIC). *vangl2* is known to function in the non-canonical Wnt/PCP signaling pathway during zebrafish gastrulation [36]. *hdac1* (rank 2 by maxIC) has been shown to regulate both the canonical and non-canonical Wnt pathways [37], particularly for oligodendrocyte specification in the CNS. *dharma* (rank 5 by simJ) is a dorsalizing transcription factor that has been shown to repress the known pathway member *bmp2b* directly [38]. Expression studies have also shown positive effects of *dharma* on *flh* (rank 23 by ICCS) expression, and a reduction of *ntl* (rank 11 by ICCS and maxIC) along the dorsal midline [39], suggesting these genes may be downstream of *dharma*.

Pathway members that were not returned as similar to *shha* warranted further investigation. For example, *sufu* was the lowest ranking pathway member, and there were multiple reasons why *sufu* was ranked as dissimilar to *shha*. The first was that only a few EQ descriptions were available for *sufu* mutants (7 total, from one genotype) and many available for *shha* mutants (77 total from 9 genotypes). While a number of the *shha* EQ descriptions were not unique, there were still a large number of annotations not-held-in-common between the two genes (see discussion below). The second reason *sufu* scored as dissimilar is because the recorded phenotype was simply different. *sufu* was annotated to inner ear, lens, and lens development, while *shha* annotations were to retina development terms, in addition to many other structures including pectoral fin, somites, brain, and muscle development terms. Because there are so few annotations to *sufu*, this was a good test for the kind of search that a researcher might perform when trying to identify candidates for interaction or further genetic study. We performed the reverse search (results in Table S24), wherein we looked for the most similar genes to *sufu* to see how the *shha* pathway members ranked. We found that *hhip* was the third most similar gene to *sufu* by simIC (and second by maxIC), as both are annotated to lens and inner ear terms. *hhip* and *sufu* are both negative-regulators of the hedgehog pathway. *kif11* is a kinesin-family member that ranked sixth most similar to*sufu*(seventh) by simIC (simJ). Although *kif11* is untested for modulating the hedgehog pathway, the fact that another family member, *kif7 (cos2)*, directly interacts with *sufu* suggests a potentially overlapping functionality between *kif7* and *kif11* based on their phenotypic profiles. So although *sufu* ranked as fairly dissimilar to *shha*, the reverse search results strengthen its membership in the hedgehog pathway, perhaps in a phenotypically distinct group of pathway members.

Some of the genes found to be highly similar to *shha* are genomically unmapped, for example *doc*. These mutants were identified in a large-scale screen wherein three phenotypic groups were described: Motility, Organs, and Mesoderm [40]. *shha* (*syu*) was identified in all three groups, while *doc* was in the Mesodermal and Motility groups. Other genes falling into the Mesoderm phenotypic group from Table 5 include *lama1* (*bal*), *ndr2* (*cyc*), *wnt11b* (*smt*), *disp1* (*con*), *gli2a* (*yot*), *prm1a* (*ubo*), *dharma* (*mom*), *scube2* (*you*), *cho*, *tbx24* (*fss*), and *chd* (*din*). *doc* scored in the top 20 most similar genes to *shha* by its simIC score. The reverse-search, using *doc* as the query against all zebrafish genes (Table S25), showed the integral-membrane protein *scube2* as the most-similar hedgehog pathway member (rank 1 by maxIC and rank 8 by simIC). The most similar genes (by all metrics) were *copb1/2*, which are known to facilitate the biosynthetic transport of *cav-1* in humans [41]. *Caveolin-1* is known to bind *Shh* for intracellular transport and to associate with *patched* in cholesterol-rich microdomains of the plasma membrane [42],[43]. That these multiple integral-membrane proteins show strong phenotypic similarity to *doc* suggests a possible role for *doc* in the hedgehog pathway, potentially as another membrane protein, or as an interacting protein. The discovery of phenotypically similar yet genomically unmapped mutations using these annotation methods and similarity algorithms is extraordinarily promising, as it suggests that the reciprocal search could provide a means of identifying candidate genes when the genetic basis of the phenotype is unknown.

#### Phenotypic similarity of orthologs

One of the ultimate goals of this methodology is to find model organism phenotypes that are similar to a human disease for which the genetic basis is unknown, thus providing candidate orthologous genes or pathway members. Therefore, our final test of our method was to determine if we could identify orthologous genes across species by comparing phenotypes alone. We found that this functionality required the UBERON ontology, which groups corresponding anatomical entities by anatomical homology, functional analogy, and structural similarity and therefore allows anatomical queries across organisms [16]. Once UBERON was included in the search algorithm, we could identify a number of orthologous genes and pathway members (Tables 6 and 7, respectively). However, the mouse and zebrafish genes most phenotypically similar to the human disease genes were not necessarily the sequence orthologs.

Investigation of the genes most similar to the human disease genes proved to be very interesting (Table 7). For example, the top three most similar genes to human *EPB41* in mouse by simIC, ICCS, and maxIC (*Epb4.9*, ICCS  = 6.01; *Epb4.1*, ICCS  = 5.94; and *Epb4.2*, ICCS  = 5.69) are all Epb family members (see Table S13 for all genes similar to *EPB41* by each metric). These three genes score as very similar because they share the highly specific phenotype spheroid erythrocyte. *Epb4.1* is linked to the human disease Elliptocytosis (EL1; OMIM#611804), and Epb4.2 is linked to Spherocytosis (SPH1; OMIM#182900). Both of these human diseases have the common cause of having a destabilized cytoskeletal scaffold of red blood cells. *Epb4.9* mutations are not linked to SPH1 or EL1 in MGI, although they may make good models because they also exhibit spheroid erythrocytes and abnormal erythrocyte lysis (MGI:2447353).

Another notable phenotype comparison is that with mouse *Cdon*, which is returned in the top four most similar genes to human *SHH* by all four metrics (simJ  = 0.24, second; simIC  = 0.12, first; ICCS  = 4.99, third; and maxIC  = 0.65, fourth). *Cdon* encodes an Ig/fibronectin repeat-containing protein that has been shown to bind to *Shh* at the cell surface and positively regulate *Shh* signaling in *Shh* expression domains in mouse [44]. *Cdon* and *SHH* mutations result in similar phenotypes such as premaxilla morphology, lip morphology, and lateral ventricle quality. *Cdon* has not yet been added to KEGG, and zebrafish *cdon* has no phenotypes annotated at this time. Based on these results, mouse *Cdon* and zebrafish *cdon* mutants may be helpful in the further analysis of the hedgehog pathway and may provide additional models of disease.

The only comparison that identified the same gene as being the most similar by all four metrics was between human *ATP2A1* and zebrafish *ryr1b*. The three most specific phenotypes these genes have in common are metal ion transmembrane transporter activity (IC  = 11.99), sarcoplasmic reticulum quality (IC  = 10.54), and muscle contraction (IC  = 5.97). *ATP2A1* is a calcium transporting ATPase that restores Ca^2+^ homeostasis following excitation of skeletal muscle. Mutations in the human gene results in Brody myopathy (OMIM #601003), which is characterized by impairment of muscular relaxation during exercise [45]. Zebrafish *ryr1b* is a calcium release channel in the sarcoplasmic reticulum involved in skeletal muscle fiber contraction [46]. *RYR1* (OMIM#180901) mutations in humans lead to congenital myopathy and multi-minicore disease (MmD), which is characterized by amorphous cores in muscle and is similar to those seen in the zebrafish *ryr1b* mutant. Therefore, because *ATP2A1* and *RYR1* are required to temporally coordinate calcium concentration, zebrafish *ryr1b* mutants might provide a useful model for Brody myopathy and MmD.

In some cases, such as for human-mouse *SOX10*, the phenotype of the ortholog appeared similar but was not returned in the top 250 when ranked by simJ (Table 6). Even though both the human disease alleles and mouse mutants had been annotated reasonably specifically to neural crest, gastrointestinal, and pigmentation terms, the sequence orthologs did not rank highly. The reason for this low similarity is because simJ and simIC penalize phenotypic profiles with many unique annotations, as was the case for *SOX10*. The use of maxIC and ICCS attempts to overcome this deficiency by examining the annotations in common. In this study, we used simJ to return the 250 most similar phenotypic profiles and then sorted the data to examine the other metrics. In the future it may be possible to incorporate maxIC and ICCS within the similarity algorithm itself to overcome this deficiency. Another reason that the sequence orthologs did not always rank very high is that some of the phenotypic data available for the orthologs were not very rich. For example, some of the phenotype annotations from ZFIN were made prior to the implementation of the EQ methodology and resulted in fairly generic EQ descriptions. Two of these generically annotated genes were not returned in the top 250 most similar genes by simJ between human and zebrafish (*atp2a1* and *fech*). We expect that, as more data are accumulated using the EQ methodology, annotation to generic nodes will comprise an increasingly smaller percentage of the total annotations.

The genes identified by this similarity algorithm are good candidates for further investigation of biological function, pathway elucidation, and identification as animal models of disease. Although some models of disease may already be in existence, the importance of having a variety of animal models for the same disease should not be underestimated. Different mutations in the same gene or in related pathway members may exhibit variable phenotypic consequences, for example lethality at different stages of development. Most importantly, our results suggest that the reciprocal search will work, where we will be able to identify animal models of human disease (or disease pathways) where the human gene is not yet known. In order to implement this, we intend to annotate the remainder of OMIM using the EQ method on OMIM phenotype synopses, and supplement the database with other disease data, to provide the necessary phenotypes for comparison. Because a mutated gene in an animal model is more readily available or identifiable, our method may hasten the identification of the genetic basis of human diseases.

### Similarity Metrics

We used three IC-based metrics to compare phenotypic profiles: simIC, ICCS, and maxIC of a pair. One non-IC-based metric, the Jaccard index (simJ), was also included in our analysis [18]. Of these metrics, ICCS has not been assessed in previous studies. To our knowledge, this is the first attempt to use any of these metrics to score similarity using composite EQ descriptions.

All metrics work in conjunction with a reasoner, thus descriptions do not have to be exact matches in order to be considered similar. The simJ metric rewards more specific matches by counting the total common subsuming descriptions over the union of all subsumed descriptions. This means that simJ is potentially open to bias in the ontology structure. We can see this if we compare the GO with the FMA—terms of comparable specificity are often located deeper in the FMA *is_a* hierarchy due to the use of high-level abstract terms in the FMA. IC-based metrics attempt to overcome biases in ontology structure by associating significance with term usage. High-level terms such as “organ” are used frequently (recall that we use the reasoner to compute indirect annotations), whereas more specific terms such as “lens” are used less frequently. Such matches for lens phenotypes are considered more significant than matches for organ phenotypes. A danger with this method is that the set of annotations may be biased, and therefore score lower than expected.

We expected IC-based metrics to fare better with the inter-species comparisons, because we have a reasonably well-sampled distribution of annotations over UBERON. There are still some biases—the zebrafish is well-suited to certain kinds of studies and mouse to others, and the literature and annotations will reflect these differences. For instance, many of the zebrafish annotations are to early developmental processes and structures because this model is well suited to developmental studies. This is a ubiquitous problem when comparing gene expression or function across species. However, it is much harder to evaluate IC-based metrics versus simJ in the context of the inter-species comparisons. If we make the assumption that orthology leads to similar phenotypes, we can use the results in Table 6 to evaluate the metrics. While the results of this study suggest that the derived IC-based metrics maxIC and ICCS may overcome some of these biases (more orthologs returned as the most similar genes), our dataset of 11 human genes does not constitute a large enough sample to statistically compare the different metrics. In the future, we aim to create a “gold standard” set of genotype-phenotype annotations that would minimize literature or experimental bias and is independently annotated by different curators to eliminate errors of commission and omission. This would allow statistical testing of sensitivity and specificity with regard to these similarity metrics. Nevertheless, our results demonstrate conclusively that one can compare phenotypes across organisms using ontology-based metrics to find biologically meaningful results. Furthermore, it is important to use multiple metrics to analyze and rank the overall similarity between genes.

### Limitations and Extensibility

The primary limitation of this method is the cost of curation from the literature, both in terms of needing domain experts as well as the time involved. There are several Natural Language Processing efforts to facilitate partial-information extraction to assist curators in identifying relevant material in the literature. For example, Textpresso [47] is able to mark up full-text literature articles for important biologically relevant terms. Adding PATO or other quality ontologies into the workflow could greatly increase the speed at which a curator could annotate the literature. However, automated tools will have errors due to terminological inaccuracy or inadequacy in published reports, and require human curatorial staff to review. This is particularly true for the human dysmorphology field, but recent efforts by a group of clinicians to standardize the terminologies used to describe human phenotypes [48] will be enormously helpful for further automated analysis. Furthermore, coordinating these standardized terminologies with the development of the Human Phenotype Ontology (HPO) [49] and in creating OMIM clinical synopses will be a necessity. The HPO was not yet available at the time of our annotation, and will be especially valuable in future cross-species phenotype studies if its development is coordinated with OMIM and the clinical dysmorphology group, and follows the OBO Foundry principles for maximal interoperability [50].

As evidenced by our evaluation of curatorial reproducibility (to be published elsewhere), ontology development is also a factor that must be considered. A fair degree of effort is required to build and maintain ontologies and the relationships between them and this effort must be informed and guided by collaborative interactions with the curators. Some domains, such as behavior, which is minimally represented in the GO, remain poorly represented by ontologies. These insufficiencies are being addressed [51],[52] and the combinatorial nature of ontologies makes new ontologies easy to add to the analysis as they become available. Another case in point are the current efforts aimed at using ontologies for image annotation (see, for example, [53] and [54]), wherein not only can the images from which the ontology terms are in part defined be easily located, but the term markup of the images themselves can be updated as the ontologies change over time.

Some key players in the zebrafish *shha* search were not included in our analysis because they were based on morpholino knockdowns rather than traditional mutants. Similarly, morpholino phenotype data from five of the 11 orthologs of the human disease genes examined were also not included in the ortholog analysis (*shha*, *sox9a*, *sox10*, *tnnt2*, and *ttna*). Future enhancements to our database structure will accommodate various mechanisms for diminishing gene function such as gene-specific morpholinos, siRNAs, or chemicals, and this will greatly expand the available dataset for comparison. Databases such as PharmGKB and the Comparative Toxicogenomics Database (CTD), both of which correlate the effects of drugs and/or toxicants to specific gene dysfunction and/or disease states [55],[56], and correlate these to specific allelic variants (PharmGKB only), might also be integrated into the system to provide additional reference data.

In order to prioritize candidate genes to be studied in the laboratory for a mutation with a defined phenotype, some combination of information is considered. The first we present here, namely the discovery of organisms with similar phenotypes in which the candidate gene may be more easily identified. However, additional information such as chromosomal position and gene expression are also often used in prioritizing candidate genes for sequencing. Since an aim of this method is to increase the efficiency in identifying candidate genes, inclusion of mapping and expression data into the workflow could further refine the search results. MODs are already using anatomy ontologies and the GO cellular component ontology for annotating both gene expression and phenotypes, and this information could be especially informative in cases where no phenotypes have been annotated to the anatomical structures in which they are expressed. In addition, recent literature suggests that much of morphological evolution is tied to mutation in cis-regulatory regions (for reviews, see [57],[58]). If it is the case that phenotypes fall into distinct classes, for example, morphological, behavioral, or physiological, then it would be interesting to see if groups of phenotypically similar genes are correlated with specific genomic or biologically relevant phenomenon. This type of contextual information can be mined from external databases (genomic, protein binding results, co-expression, etc.) and would not only facilitate candidate gene prioritization but may also provide insight as to the molecular basis of gene evolution.

Another biologically interesting question we considered was whether zebrafish paralogs would have combined phenotypic profiles that are complementary in toto to their mammalian ortholog. An interesting feature of zebrafish is that they had a genome-wide duplication, which occurred as part of the *teleost* radiation approximately 350 million years ago, and some of the duplicated genes persist in the modern zebrafish genome [59]. The occurrence of two orthologs in zebrafish of a single mammalian gene provides a unique opportunity to examine the degree to which the phenotypes of mutations in these paralogs are similar or complementary. It is well known that a number of paralogs have diverged so as to become complementary or expanded in their expression patterns and/or functions, whereas others are redundant or nonfunctional [60],[61]. In many cases only one of a pair of paralogs has been studied by mutational analysis, but the other has been studied using morpholino knockdown reagents. Therefore, the analysis of phenotypic similarity between paralogs will also be facilitated by the future inclusion of the knockdown phenotypes into our dataset.

A project that relates and extends this work is the Phenoscape [62] project, which uses ontologies and the EQ method to record evolutionarily variable morphological characters for a large clade of fishes. This group has been very successful in having the comparative morphology community annotate evolutionary phenotypes. The goal is to use these explicitly recorded character states to query MODs for similar phenotypes, thus gaining candidate genes for evolutionary change. It will be interesting to utilize the phenotypic similarity of related species as an added component to the methodology presented here. Both approaches could well inform one another, providing a better understanding of the evolution of signaling pathways and anatomical form.

In this study, we show that by using ontologies for phenotype annotation, one can precisely record and quantify similar phenotypes. Annotation of phenotypes using the EQ method will not only facilitate the use of a common language necessary for comparing phenotypes, it will also facilitate the identification of genotypes with similar phenotypes within and across species, providing candidate genes for human disease, evolutionary change, and pathway characterization.

## Materials and Methods

### OMIM Statistics

Statistics for free-text query of OMIM records were obtained on 2/6/2009 (Table 1). Statistics for the number of OMIM gene records with associated phenotypes were obtained by doing a query in OMIM for any gene record (* or +) with a filter selecting records with allelic variant descriptions and/or clinical synopses. Statistics for the percentage of OMIM phenotype/disease records with known molecular genetic basis were derived from the table of OMIM statistics at http://www.ncbi.nlm.nih.gov/Omim/mimstats.html, by dividing the count for records with a “Phenotype description, molecular basis known” by the total number of phenotype records (statistics are as of 8/10/2009).

### Selection of Genes/Records for Annotation

Human genes from OMIM were selected first by ranking by those with known and described mutant homologs in *Danio rerio* and *Drosophila melanogaster*, then by having the greatest number of detailed descriptions of alleles in OMIM. We selected the following 11 genes to be annotated from their OMIM record: *ATP2A1* (108730), *EPB41* (130500), *EXT2* (608210), *EYA1* (601653), *FECH* (177000), *PAX2* (167409), *SHH* (600725), *SOX9* (608160), *SOX10* (602229), *TNNT2* (191045), and *TTN* (188840). *EYA1*, *PAX2*, *SOX9*, *SOX10*, and *TTN* were selected for recording by three independent curators to test for annotation consistency (to be published elsewhere). Where an OMIM gene record referred to a disease record, the annotators would capture as much general phenotype information about that disease as possible.

### Annotation Software and Storage

We write ontology terms prefixed with the name of the ontology; abbreviations are provided at the beginning of this paper. We use ZFA:gut in place of ZFA:0000112 for legibility purposes. The actual computationally parseable form would use the numeric IDs.

All OMIM annotations were created with Phenote [24] software, using the “human” configuration. This included the following ontologies: CL, CHEBI, FMA, GO, and EDHAA for entity selection, and PATO for quality selection. All annotations were recorded with provenance assigned to the PubMed identifier (PMID) for the original publication as listed in the OMIM record. Ontologies were updated daily during annotation, and any annotations to obsolete terms were reconciled prior to analysis. Annotations, together with reference ontologies, that were analyzed for this paper can be found at the stable URL: http://obo.svn.sourceforge.net/viewvc/obo/phenotype-commons/annotations/OMIM/archive/2009/.

### Additional Annotation Sources

Additional phenotype annotations were retrieved for cross-species comparison from MGI [33], ZFIN [13], GAD [63], NCBI gene [64], and homologene [65] in September 2008. Ontologies used in the analysis were downloaded from the OBO Foundry repository [66] in August 2008: BP-XP-UBERON (December 2008), ChEBI, CL, DO, DO-XP-FMA, EDHAA, FMA, GO-BP, GO-CC, GO-MF, MA, MP-XP, PATO, SO, UBERON, ZFA, and ZFS. To link cross-species annotations made to species-specific anatomy ontologies (ssAOs), we created an “Uber-ontology,” UBERON, to fill the gap between the general Common Anatomy Reference Ontology (CARO) [67] and the ssAOs. The first version of UBERON was generated automatically by aligning existing ssAOs and anatomical reference ontologies, and then partially manually curated. Ontologies referenced include: FMA, MA, EHDAA, ZFA, TAO, NIF, GAID, CL, XAO, MAT, FBbt, AAO, BILA, WBbt, and CARO. Additional details can be found in [17] and [16]. All ontologies were loaded into OBD, together with the annotations from the sources listed in Table 4.

### Reasoning

Reasoning was performed over the combined set of annotations, ontologies, and ontology mappings. We used the OBD RuleBasedReasoner to compute the closure of transitive relations and to compute inferred subsumption relationships between EQ descriptions [28].

### Analysis

The phenotype analysis was performed using the OBD System [28] that implements a number of similarity metrics, described as follows. All similarity metrics are based on the reasoned graph, and annotations are propagated up the subsumption hierarchy.

Most of these metrics use the IC (Equation 1) of a term or EQ phenotype (collectively called a description), which is the negative log of the probability of that description being used to annotate a gene, allele, or genotype (collectively called a feature). 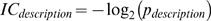
where the probability of a description is the number of features annotated with that description over the total number of features in the database (Equation 2):
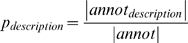



Here *annot_description_* denotes the number of features to which the description applies, after reasoning has been performed. This means that very general descriptions, such as “morphology of anatomical structure,” which subsume many more specific descriptions, are applicable to a greater number of features and thus have a low IC.

### maxIC

The maxIC is obtained by taking all descriptions shared by a pair of features and finding the description(s) with the highest IC. This may be an exact match, or it may be a subsuming description inferred by the reasoner. One characteristic of the maxIC score is that it can hide the contributions of annotations not in the maxIC set. This score is equivalent to the “maximum” variant of the Resnick similarity, as described in [18].

### ICCS

This metric attempts to match every description directly annotated in one feature with a directly annotated description in the other feature. Each directly annotated description *d_i_* is compared against all the descriptions *d’_1_*, *d’_2_*,…in the other feature being compared. The most specific (highest scoring) common subsuming description is found, and the unique set of these is called the common subsumers. The ICCS is the average IC of all the common subsumers in this unique set.

This measure is shown in Figure 4 where the center triptych shows the common subsumers. The ICCS metric is described in [28] and has not been described previously to our knowledge. It can be considered a composition of the average and maximum Resnick measures as described in [18].

### simIC

Given two phenotypic profiles, for example the phenotypic profiles of two genes, or two genotypes, or the two profiles generated by two curators annotating the same genotype, we can calculate the sum of the IC scores for (a) those phenotype EQ descriptions that are held in common (the intersection) and (b) the combined total set of phenotype EQ descriptions (the union). Looking at the ratio of these two sums (those that are shared versus the totality), we can obtain a measure of how similar the two phenotypic profiles are, with perfectly identical phenotypes having a score of 1. The simIC measure is illustrated in (Equation 3).
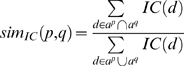



Here *a^p^* denotes the total set of descriptions that can be applied to *p*, including subsuming descriptions. As an example, given two genotypes, *p* and *q*, the simIC is obtained by dividing the sum of ICs for all descriptions in common by the sum of all descriptions in the union. Here, descriptions include the actual descriptions used in the profile, and all subsuming descriptions as determined by the reasoner. This metric penalizes nodes that have differing annotations.

### simJ

We used one additional similarity metric, the simJ, which does not utilize the IC measures. The simJ between two profiles is the ratio between the number of descriptions in common versus the number of descriptions in both profiles. This is also called the “Jaccard index” or the “Jaccard similarity coefficient.” The number of descriptions in common is called simTO in [18]. The simJ (Equation 4) is a variant of the normalized simTO:
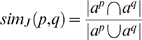



### Gene Comparisons

Note that for comparisons between two genes, all annotations made to heterozygous and homozygous genotypes were first propagated to the single (or both, if known) alleles, and then propagated to their gene parent. The genotype annotations used in each query were excluded from the background set in calculating the overall score (Figure 5).

For the allele-to-allele comparisons, we calculated each metric for all pairwise combinations of alleles. Similarity scores between a pair of alleles were sorted into intra-gene (same gene) and inter-gene (different genes) sets, and the mean scores for each gene compared. The significance of the difference between the mean scores for each gene was calculated using a two-tailed Student's *t*-test.

For the zebrafish *shha* query, we also compared this gene against all other zebrafish genes (2,908 genes in the total set). For the inter-species queries, we exhaustively compared each gene against all other genes using simJ and then computed all metrics on the top 250.

## Supporting Information

Table S1 Comparison between zebrafish *shha* and all currently annotated zebrafish genes. Reported is an expanded list of what is reported in Table 5, including ranks and scores for each metric (simIC, simJ, avgICCS, and maxIC) and phenotype giving the maxIC score. Known pathway genes are highlighted in yellow, and others reported in the text are highlighted in blue. Only the first 1,000 ranks are calculated.
(0.79 MB XLS)

Table S2 Comparison between human *ATP2A1* and zebrafish genes. The top 250 genes (by simJ) are reported, together with the rank and score for each metric (simIC, simJ, avgICCS, and maxIC). Additionally, the maxIC phenotype is reported using ontology identifiers. See Materials and Methods for a list of ontology prefixes. Known sequence orthologs are highlighted in yellow and listed in Table 6, if present. Genes at rank 1 are listed in Table 7.
(0.08 MB XLS)

Table S3 Comparison between human *EPB41* and zebrafish genes. The top 250 genes (by simJ) are reported, together with the rank and score for each metric (simIC, simJ, avgICCS, and maxIC). Additionally, the maxIC phenotype is reported using ontology identifiers. See Materials and Methods for a list of ontology prefixes. Known sequence orthologs are highlighted in yellow and listed in Table 6, if present. Genes at rank 1 are listed in Table 7.
(0.08 MB XLS)

Table S4 Comparison between human *EXT2* and zebrafish genes. The top 250 genes (by simJ) are reported, together with the rank and score for each metric (simIC, simJ, avgICCS, and maxIC). Additionally, the maxIC phenotype is reported using ontology identifiers. See Materials and Methods for a list of ontology prefixes. Known sequence orthologs are highlighted in yellow and listed in Table 6, if present. Genes at rank 1 are listed in Table 7.
(0.09 MB XLS)

Table S5 Comparison between human *EYA1* and zebrafish genes. The top 250 genes (by simJ) are reported, together with the rank and score for each metric (simIC, simJ, avgICCS, and maxIC). Additionally, the maxIC phenotype is reported using ontology identifiers. See Materials and Methods for a list of ontology prefixes. Known sequence orthologs are highlighted in yellow and listed in Table 6, if present. Genes at rank 1 are listed in Table 7.
(0.09 MB XLS)

Table S6 Comparison between human *FECH* and zebrafish genes. The top 250 genes (by simJ) are reported, together with the rank and score for each metric (simIC, simJ, avgICCS, and maxIC). Additionally, the maxIC phenotype is reported using ontology identifiers. See Materials and Methods for a list of ontology prefixes. Known sequence orthologs are highlighted in yellow and listed in Table 6, if present. Genes at rank 1 are listed in Table 7.
(0.08 MB XLS)

Table S7 Comparison between human *PAX2* and zebrafish genes. The top 250 genes (by simJ) are reported, together with the rank and score for each metric (simIC, simJ, avgICCS, and maxIC). Additionally, the maxIC phenotype is reported using ontology identifiers. See Materials and Methods for a list of ontology prefixes. Known sequence orthologs are highlighted in yellow and listed in Table 6, if present. Genes at rank 1 are listed in Table 7.
(0.09 MB XLS)

Table S8 Comparison between human *SHH* and zebrafish genes. The top 250 genes (by simJ) are reported, together with the rank and score for each metric (simIC, simJ, avgICCS, and maxIC). Additionally, the maxIC phenotype is reported using ontology identifiers. See Materials and Methods for a list of ontology prefixes. Known sequence orthologs are highlighted in yellow and listed in Table 6, if present. Genes at rank 1 are listed in Table 7.
(0.09 MB XLS)

Table S9 Comparison between human *SOX10* and zebrafish genes. The top 250 genes (by simJ) are reported, together with the rank and score for each metric (simIC, simJ, avgICCS, and maxIC). Additionally, the maxIC phenotype is reported using ontology identifiers. See Materials and Methods for a list of ontology prefixes. Known sequence orthologs are highlighted in yellow and listed in Table 6, if present. Genes at rank 1 are listed in Table 7.
(0.09 MB XLS)

Table S10 Comparison between human *SOX9* and zebrafish genes. The top 250 genes (by simJ) are reported, together with the rank and score for each metric (simIC, simJ, avgICCS, and maxIC). Additionally, the maxIC phenotype is reported using ontology identifiers. See Materials and Methods for a list of ontology prefixes. Known sequence orthologs are highlighted in yellow and listed in Table 6, if present. Genes at rank 1 are listed in Table 7.
(0.09 MB XLS)

Table S11 Comparison between human *TNNT2* and zebrafish genes. The top 250 genes (by simJ) are reported, together with the rank and score for each metric (simIC, simJ, avgICCS, and maxIC). Additionally, the maxIC phenotype is reported using ontology identifiers. See Materials and Methods for a list of ontology prefixes. Known sequence orthologs are highlighted in yellow and listed in Table 6, if present. Genes at rank 1 are listed in Table 7.
(0.08 MB XLS)

Table S12 Comparison between human *TTN* and zebrafish genes. The top 250 genes (by simJ) are reported, together with the rank and score for each metric (simIC, simJ, avgICCS, and maxIC). Additionally, the maxIC phenotype is reported using ontology identifiers. See Materials and Methods for a list of ontology prefixes. Known sequence orthologs are highlighted in yellow and listed in Table 6, if present. Genes at rank 1 are listed in Table 7.
(0.09 MB XLS)

Table S13 Comparison between human *ATP2A1* and mouse genes. The top 250 genes (by simJ) are reported, together with the rank and score for each metric (simIC, simJ, avgICCS, and maxIC). Additionally, the maxIC phenotype is reported using ontology identifiers. See Materials and Methods for a list of ontology prefixes. Known sequence orthologs are highlighted in yellow and listed in Table 6, if present. Genes at rank 1 are listed in Table 7.
(0.08 MB XLS)

Table S14 Comparison between human *EPB41* and mouse genes. The top 250 genes (by simJ) are reported, together with the rank and score for each metric (simIC, simJ, avgICCS, and maxIC). Additionally, the maxIC phenotype is reported using ontology identifiers. See Materials and Methods for a list of ontology prefixes. Known sequence orthologs are highlighted in yellow and listed in Table 6, if present. Genes at rank 1 are listed in Table 7.
(0.08 MB XLS)

Table S15 Comparison between human *EXT2* and mouse genes. The top 250 genes (by simJ) are reported, together with the rank and score for each metric (simIC, simJ, avgICCS, and maxIC). Additionally, the maxIC phenotype is reported using ontology identifiers. See Materials and Methods for a list of ontology prefixes. Known sequence orthologs are highlighted in yellow and listed in Table 6, if present. Genes at rank 1 are listed in Table 7.
(0.08 MB XLS)

Table S16 Comparison between human *EYA1* and mouse genes. The top 250 genes (by simJ) are reported, together with the rank and score for each metric (simIC, simJ, avgICCS, and maxIC). Additionally, the maxIC phenotype is reported using ontology identifiers. See Materials and Methods for a list of ontology prefixes. Known sequence orthologs are highlighted in yellow and listed in Table 6, if present. Genes at rank 1 are listed in Table 7.
(0.09 MB XLS)

Table S17 Comparison between human *FECH* and mouse genes. The top 250 genes (by simJ) are reported, together with the rank and score for each metric (simIC, simJ, avgICCS, and maxIC). Additionally, the maxIC phenotype is reported using ontology identifiers. See Materials and Methods for a list of ontology prefixes. Known sequence orthologs are highlighted in yellow and listed in Table 6, if present. Genes at rank 1 are listed in Table 7.
(0.08 MB XLS)

Table S18 Comparison between human *PAX2* and mouse genes. The top 250 genes (by simJ) are reported, together with the rank and score for each metric (simIC, simJ, avgICCS, and maxIC). Additionally, the maxIC phenotype is reported using ontology identifiers. See Materials and Methods for a list of ontology prefixes. Known sequence orthologs are highlighted in yellow and listed in Table 6, if present. Genes at rank 1 are listed in Table 7.
(0.08 MB XLS)

Table S19 Comparison between human *SHH* and mouse genes. The top 250 genes (by simJ) are reported, together with the rank and score for each metric (simIC, simJ, avgICCS, and maxIC). Additionally, the maxIC phenotype is reported using ontology identifiers. See Materials and Methods for a list of ontology prefixes. Known sequence orthologs are highlighted in yellow and listed in Table 6, if present. Genes at rank 1 are listed in Table 7.
(0.08 MB XLS)

Table S20 Comparison between human *SOX10* and mouse genes. The top 250 genes (by simJ) are reported, together with the rank and score for each metric (simIC, simJ, avgICCS, and maxIC). Additionally, the maxIC phenotype is reported using ontology identifiers. See Materials and Methods for a list of ontology prefixes. Known sequence orthologs are highlighted in yellow and listed in Table 6, if present. Genes at rank 1 are listed in Table 7.
(0.08 MB XLS)

Table S21 Comparison between human *SOX9* and mouse genes. The top 250 genes (by simJ) are reported, together with the rank and score for each metric (simIC, simJ, avgICCS, and maxIC). Additionally, the maxIC phenotype is reported using ontology identifiers. See Materials and Methods for a list of ontology prefixes. Known sequence orthologs are highlighted in yellow and listed in Table 6, if present. Genes at rank 1 are listed in Table 7.
(0.09 MB XLS)

Table S22 Comparison between human *TNNT2* and mouse genes. The top 250 genes (by simJ) are reported, together with the rank and score for each metric (simIC, simJ, avgICCS, and maxIC). Additionally, the maxIC phenotype is reported using ontology identifiers. See Materials and Methods for a list of ontology prefixes. Known sequence orthologs are highlighted in yellow and listed in Table 6, if present. Genes at rank 1 are listed in Table 7.
(0.08 MB XLS)

Table S23 Comparison between human *TTN* and mouse genes. The top 250 genes (by simJ) are reported, together with the rank and score for each metric (simIC, simJ, avgICCS, and maxIC). Additionally, the maxIC phenotype is reported using ontology identifiers. See Materials and Methods for a list of ontology prefixes. Known sequence orthologs are highlighted in yellow and listed in Table 6, if present. Genes at rank 1 are listed in Table 7.
(0.08 MB XLS)

Table S24 Comparison between zebrafish *sufu* and all currently annotated zebrafish genes. Ranks and scores for each metric (simIC, simJ, avgICCS, and maxIC) and phenotype giving the maxIC score are reported. Known *shh* pathway genes are highlighted in yellow. Only ranks for first 1,000 are calculated.
(0.25 MB XLS)

Table S25 Comparison between zebrafish *doc* and all currently annotated zebrafish genes. Ranks and scores for each metric (simIC, simJ, avgICCS, and maxIC) and phenotype giving the maxIC score are reported. Known *shh* pathway genes are highlighted in yellow. Only ranks for first 1,000 are calculated.
(0.27 MB XLS)

## Abbreviations

ATP2A1: ATPase
Ca(2+): Transporting, Fast-Twitch 1
CHEBI: Chemical Entities of Biological Interest Ontology
CL: Cell Type Ontology
CTD: Comparative Toxicogenomics Database
DO: Disease Ontology
EHDAA: Human developmental anatomy, abstract version
ENVO: Environment Ontology
EPB41: ERYTHROCYTE MEMBRANE PROTEIN BAND 4.1
EXT2: Exostosin 2
EYA1: Eyes Absent 1 gene
FB: FlyBase
FBbt: FlyBase Anatomy Ontology
FECH: FERROCHELATASE
FMA: Foundational Model of Anatomy
GAD: Gene Association Database
GC: Genetic context ontology
GO: Gene Ontology
HPO: Human Phenotype Ontology
ICCS: Information Content of the Common Subsumer
LBNL: Lawrence Berkeley National Laboratory
MGI: Mouse Genome Informatics
MmD: multi-minicore disease
MP: Mammalian Phenotype ontology
NCBI: National Center for Biotechnology Information
OBD: Open Biomedical Database
OBOF: Open Biomedical Ontologies Format
OMIM: Online Mendelian Inheritance in Man
OWL: Web Ontology Language
OWL-DL: OWL Description Logic
PATO: Phenotype and Trait Ontology
PAX2: Paired Box Gene 2
PharmGKB: The Pharmacogenetics and Pharmacogenomics Knowledge Base
PMID: PubMed Identifier
REST: REpresentational State Transfer
SHH: Sonic HedgeHog
simJ: Jaccard similarity coefficient
SOX9: SRY-box 9 gene
SOX10: SRY-box 10 gene
ssAOs: species-specific anatomy ontologies
TNNT2: Troponin T 2
TTN: Titin
ZFA: Zebrafish Anatomy
ZFIN: Zebrafish Information Network
ZFS: Zebrafish Stage Ontology
